# Early detection of SARS-CoV-2 variants through dynamic co-mutation network surveillance

**DOI:** 10.3389/fpubh.2023.1015969

**Published:** 2023-01-23

**Authors:** Qiang Huang, Huining Qiu, Paul W. Bible, Yong Huang, Fangfang Zheng, Jing Gu, Jian Sun, Yuantao Hao, Yu Liu

**Affiliations:** ^1^Department of Medical Statistics, School of Public Health, Sun Yat-sen University, Guangzhou, China; ^2^Guangdong Artificial Intelligence Machine Vision Engineering Technology Research Center, Guangzhou, China; ^3^College of Arts and Sciences, Marian University, Indianapolis, IN, United States; ^4^Institute of Public Health, Guangzhou Medical University & Guangzhou Center for Disease Control and Prevention, Guangzhou, China; ^5^School of Traditional Chinese Medicine Healthcare, Guangdong Food and Drug Vocational College, Guangzhou, China; ^6^Department of Clinical Research, The Third Affiliated Hospital of Sun Yat-sen University, Guangzhou, China; ^7^Peking University Center for Public Health and Epidemic Preparedness & Response, Beijing, China

**Keywords:** SARS-CoV-2, co-mutation, surveillance, network, community detection

## Abstract

**Background:**

Precise public health and clinical interventions for the COVID-19 pandemic has spurred a global rush on SARS-CoV-2 variant tracking, but current approaches to variant tracking are challenged by the flood of viral genome sequences leading to a loss of timeliness, accuracy, and reliability. Here, we devised a new co-mutation network framework, aiming to tackle these difficulties in variant surveillance.

**Methods:**

To avoid simultaneous input and modeling of the whole large-scale data, we dynamically investigate the nucleotide covarying pattern of weekly sequences. The community detection algorithm is applied to a co-occurring genomic alteration network constructed from mutation corpora of weekly collected data. Co-mutation communities are identified, extracted, and characterized as variant markers. They contribute to the creation and weekly updates of a community-based variant dictionary tree representing SARS-CoV-2 evolution, where highly similar ones between weeks have been merged to represent the same variants. Emerging communities imply the presence of novel viral variants or new branches of existing variants. This process was benchmarked with worldwide GISAID data and validated using national level data from six COVID-19 hotspot countries.

**Results:**

A total of 235 co-mutation communities were identified after a 120 weeks' investigation of worldwide sequence data, from March 2020 to mid-June 2022. The dictionary tree progressively developed from these communities perfectly recorded the time course of SARS-CoV-2 branching, coinciding with GISAID clades. The time-varying prevalence of these communities in the viral population showed a good match with the emergence and circulation of the variants they represented. All these benchmark results not only exhibited the methodology features but also demonstrated high efficiency in detection of the pandemic variants. When it was applied to regional variant surveillance, our method displayed significantly earlier identification of feature communities of major WHO-named SARS-CoV-2 variants in contrast with Pangolin's monitoring.

**Conclusion:**

An efficient genomic surveillance framework built from weekly co-mutation networks and a dynamic community-based variant dictionary tree enables early detection and continuous investigation of SARS-CoV-2 variants overcoming genomic data flood, aiding in the response to the COVID-19 pandemic.

## Introduction

The evolution of severe acute respiratory syndrome coronavirus 2 (SARS-CoV-2) presents ongoing risks and threats to natural and vaccine-induced immunity and the effectiveness of diagnostics and therapeutics (1–3). With the rapidly increasing volume of SARS-CoV-2 genomes, leveraging this wealth of data for variant surveillance quickly becomes intractable due to a daunting computational hurdle of using gold-standard phylogenetic approaches (4). Routine analysis of the expanding scale of sequence data helps the authorities to detect and monitor variant viruses for further characterization and assessment of risk but developing efficient methods is still a core need in this field.

A growing body of evidence shows that multiple mutations arising simultaneously in one genome, referred as co-mutation, can be a reliable predictor for viral variant monitoring (5–9). A collection and combination of co-mutation communities resulted from genomic data accumulated over time helps to capture the evolution and transmission patterns of SARS-CoV-2 (7). Nevertheless, the efficacy of periodic surveillance of co-mutation-based SARS-CoV-2 phylogeny using only updated data for a more computational feasible but globally correspondent evolutionary profile is still an outstanding issue to be addressed.

In this study, we developed a co-mutation network surveillance framework to dynamically scout the nucleotide co-occurring pattern of sequences retrieved weekly. The homogeneous co-mutations in the network were found to agglomerate into groups of co-mutation communities characterized as variant markers. These variant markers contribute to weekly updates of a dictionary tree representing community-based SARS-CoV-2 evolution. Emerging communities indicate the presence of new viral variants or new branches of existing variants. We demonstrate this process and interpretation through dynamic creation of global evolution history of major SARS-CoV-2 variants and validate its variant surveillance efficiency by tracking multiple variants circulating in some of the major contributors that provide SARS-CoV-2 genomes in “Global Initiative on Sharing Avian Influenza Data” (GISAID) (10).

## Materials and methods

### Data source

A total of 11,529,602 SARS-CoV-2 genomes were retrieved from GISAID on 25 June 2022. The low coverage sequences (genomes with >5% Ns) were first excluded and only complete genomes (genome length >29,000 base pairs) sampled from humans with explicit collection dates were included. Genomes with duplicated GISAID sequence names were further detected and eliminated, resulting in a dataset of 10,249,122 (88.9%) records. Due to sparse or delayed sequence submission during early epidemic and at the end of data retrieval, we exclusively involved genomes sampled between 1 March 2020 and 18 June 2022 in our study. Then a bioinformatic pipeline, as reported by our previous study (9), was applied to the remaining 10,246,539 (88.9%) sequences to extract and annotate all single nucleotide polymorphisms (SNPs) and insertions/deletions (INDELs) for each genome. In consequence, 519,230,825 mutational events from these sequences were exported and labeled with the sampling week. Since the earliest sampling time in this study was 1 March 2020, the 1^st^ week was defined as from 1 to 7 March 2020. And the last week of the study period was designated from 12 to 18 June 2022, i.e., the 120^th^ week.

### Co-mutation network surveillance

**Figure 1:**
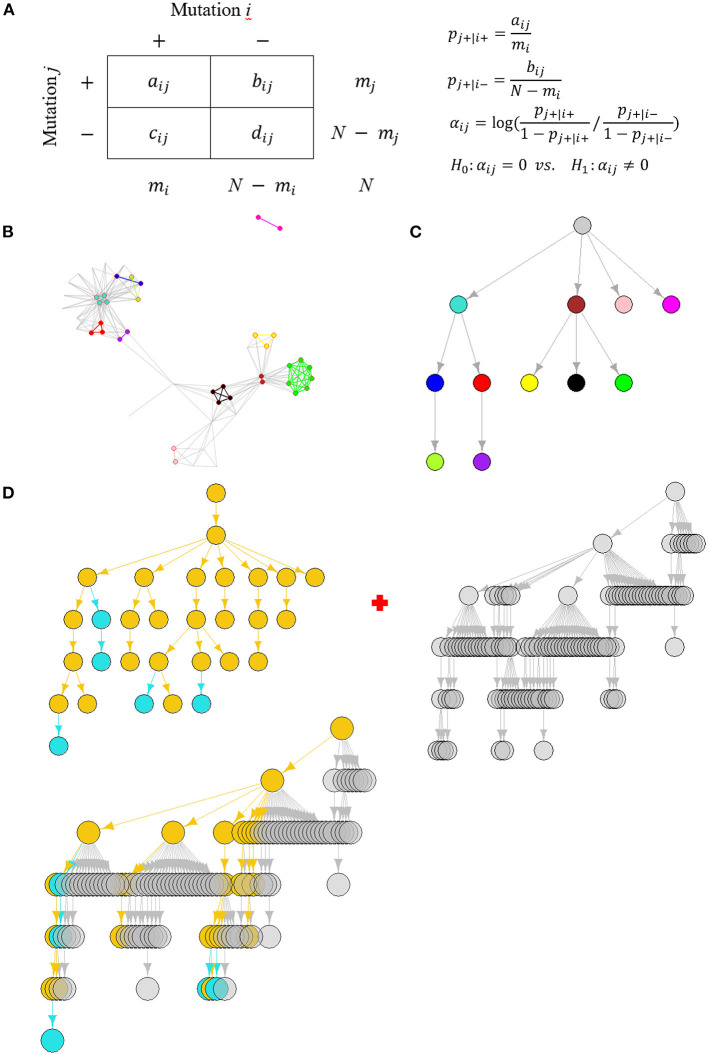
The schema of dynamic SARS-CoV-2 co-mutation network surveillance. **(A)** The affinity-model-based identification of co-mutation pairs. **(B)** An illustration of weekly co-mutation network, where nodes and edges with the same colors represent the gathering homogeneous co-mutations, referred to as co-mutation communities. **(C)** The arborescence indicating SARS-CoV-2 evolution through modeling the hierarchical containment of partition of viral population based on the detected communities' presence or not, where nodes correspond to communities with the same colors as in **(B)**. **(D)** Weekly updates of a dictionary tree representing community-based SARS-CoV-2 evolution, which is a “union” of two trees. One is the co-mutation community tree detected at the current week, where historically circulating communities were colored in yellow but emerging communities in cyan. Another is last week's dictionary tree whose nodes are colored in gray. The union results in an update of the dictionary tree where nodes and edges included in at least one tree are preserved and colored by their circulating features.

SARS-CoV-2 variant surveillance are performed periodically. We repeatedly executed weekly detection protocols for real-time tracking of circulating co-mutation network using our method (Figures 1A–C). These co-mutation networks across weeks were integrated to form a dynamic dictionary for variant monitoring and early warning (Figure 1D). The following subsections detail the complete workflow.

Before network creation, mutations with an allele frequency at the weekly level ≤1% were eliminated since such mutations are considered unfixed in a viral population (11) leading to poor computation effectiveness in co-mutation community detection (7).

### Step 1. Weekly co-mutation community network

#### Step 1.1. The affinity model for identification of paired co-mutations

We model a mutation's tendency to be present or absent in a genome where another mutation is already present (Figure 1A). Suppose that, at each genome, independently of all others, mutation *j* is present with probability *p*_*j*+|*i*+_ if mutation *i* is present but with probability *p*_*j*+|*i*−_ if mutation *i* is absent. Their tendency to co-occur can be defined as the degree of difference of the two probabilities using a log odds ratio (12).


(1) $$\begin{array}{l} \alpha_{ij}=\log\left(\frac{p_{j+|i+}}{1-p_{j+|i+}}/\frac{p_{j+|i-}}{1-p_{j+|i-}}\right). \end{array}$$

When paired mutations co-occur more often, the log odds ratio is expected to be positive (Supplementary Figure 1A). Or, conversely, their log odds ratio becomes negative (Supplementary Figure 1B). A more or less equal value of *p*_*j*+|*i*+_ and *p*_*j*+|*i*−_ turns α_*ij*_ to be close to zero, suggesting mutation *j*'s presence or absence is independent to mutation *i* (Supplementary Figure 1C). Then, identification of co-mutation pairs becomes a series of hypothesis testing problems with *H*_0_:α_*ij*_ = 0 (Figure 1A). Our analysis considered only co-mutations with positive co-occurrence.

It has been shown that the binary co-occurrence *X* follows the extended hypergeometric distribution with a general form of,


(2) $$\begin{array}{l} P\left(X=k\right)=\left(\begin{array}{c} m_{i}\\ k \end{array}\right)\left(\begin{array}{c} {N-m}_{i}\\ m_{j}-k \end{array}\right)e^{\alpha_{ij}k}/\overset{m_{j}}{\underset{s=0}{\sum }}\left(\begin{array}{c} m_{i}\\ s \end{array}\right)\left(\begin{array}{c} {N-m}_{i}\\ m_{j}-s \end{array}\right)e^{\alpha_{ij}s} \end{array}$$

for max(*m*_*i*_ + *m*_*j*_ − *N*, 0) ≤ *k* ≤ min(*m*_*i*_, *m*_*j*_) and the same co-mutation distribution arises if their roles are switched (12). Obviously, this distribution (i.e., Eq. 2) depends only on *m*_*i*_, *m*_*j*_, *N* and α_*ij*_, but not on *p*_*j*+|*i*+_ or *p*_*j*+|*i*−_, indicating insensitive to their respective prevalence. The α_*ij*_ can be estimated by maximizing Eq. 2 with *X*= “observed amount of co-occurrence of mutation *i* and *j*” substituted for *k* and the maximum likelihood estimate ${\hat{\alpha }}_{ij}$ is termed to be an affinity metric of co-occurrence (12). Then the *P*-values can be calculated as the exact probabilities of co-occurrence greater than or less than what is observed. The computation of false discovery rate across all *P*-values provides correction for multiple hypothesis testing and the cutoff for identification of paired co-mutations is set at 0.001.

#### Step 1.2. Co-mutation network and co-mutation communities

Each pair of co-mutations will result in a connection or an edge leading to an adjacency matrix which defines the co-mutation network. Let $A^{w}=\left(A_{ij}^{w}\right)$ be the adjacency matrix where $A_{ij}^{w}=1$ if mutation *i* and *j* form a co-mutation pair at week *w*, or else $A_{ij}^{w}=0$. Specifically, $A_{ij}^{w}=0$ if *i* = *j*. So, it defines an undirected network (Figure 1B), denoted by *G*_*w*_ = (*V*_*w*_, *E*_*w*_), where *V*_*w*_ is a set of nodes corresponding to all mutations involved at week *w* and *E*_*w*_ is a set of edges each linking a co-mutation pair.

The affinity model indiscriminately identifies homogeneous and heterogeneous co-mutation pairs (Supplementary Figures 1D, E), which are respectively abbreviated as HoCPs and HeCPs. A HoCP is a pair of co-occurring mutations with equal or close mutation frequencies, while a HeCP is the opposite. A lot of indexes can be used to measure the homogeneity of paired co-mutations. For simplicity, we inherited the rate of the co-mutation (RCM) from Qin et al. (7) to determine a HoCP.


(3) $$\begin{array}{l} RCM_{ij}=\frac{\left|M_{i}\cap M_{j}\right|}{\sqrt{{|M}_{i}|\cdot |M_{j}|}} \end{array}$$

where *M*_*t*_ = {*Genomes with mutation t*} (*t* = *i or j*) and |·| denotes the total number of elements in the set. This is equivalent to the Ochiai efficient (13), which ranges from 0 to 1. The larger it is, the more homogeneous the two mutations co-occur in the same viral population. Due to the sequencing errors, a relaxed RCM 0.9 instead of 1.0 was empirically used as a cutoff to determine a HoCP (Supplementary Figures 2A, B).

The HoCPs identified form an aggregated community structure with groups of strongly linked nodes (Figure 1B). We excluded non-HoCP nodes and applied the Girvan-Newman partition algorithm (14) to discover these HoCP groups, named with co-mutation communities hereafter, which was executed by R igraph (15) package. Different from co-mutation modules defined by shared co-mutations (7), the community detection method may get finer division for these HoCPs (Supplementary Figures 3A, B).

#### Step 1.3. Weekly co-mutation community tree

The co-mutation communities exhibit hierarchical organization in weekly co-mutation network (Figure 1B). This hierarchy can be captured by division of the viral genomes and their hierarchical containment according to the detected communities' presence or not (7). We built an arborescence, a directed rooted tree, to depict their concatenated containment between these divisions and then used its topological ordering to find the hierarchical relationship (Figure 1C).

In detail, the arborescence, denoted by *T*_*w*_ = (*C*_*w*_, *R*_*w*_, *r*), incorporates nodes *C*_*w*_ corresponding to genome groups present and labeled with the detected co-mutation communities at week *w*, joint by directed edges *R*_*w*_ representing the identified containing relationships with the direction going from parent to child and rooted by a complete group *r* (∈*C*_*w*_) including all genomes besides those with absence of any co-mutation community. Different from the exact containing relationship, some of the genomes in a child set may not be included in its parent set due to sequencing errors or algorithm limitation in genotype or mutation calling. To that end, we defined a containing relationship *c*_*x*_ ⊂ *c*_*y*_ (*c*_*x*_, *c*_*y*_ ∈ *C*_*w*_) through their Simpson index beyond a cutoff determined by evaluation of historical communities (Supplementary Figure 4). The Simpson similarity is calculated as,


(4) $$\begin{array}{l} Sim_{{c_{x}c}_{y}}=\frac{\left|c_{x}\cap c_{y}\right|}{\min\left(\left|c_{x}|,\;|c_{y}\right|\right)}. \end{array}$$

It ranges from 0 to1 with a value of 1 representing that all elements in a child set are included in its parent set. That is to say, *c*_*x*_ ⊂ *c*_*y*_ if and only if most of the elements in *c*_*x*_ are elements in *c*_*y*_ where |*c*_*x*_| < |*c*_*y*_|. To model evolution histories of SARS-CoV-2 similar to a phylogenetic tree, we constrained edges in *R*_*w*_ to those resulting from direct containing relationships. For example, if the concatenated containing relationship *c*_*x*_ ⊂ *c*_*y*_ ⊂ *c*_*z*_ is found, only *c*_*x*_ ⊂ *c*_*y*_ and *c*_*y*_ ⊂ *c*_*z*_ but not *c*_*x*_ ⊂ *c*_*z*_ will be included, resulting in *c*_*z*_ → *c*_*y*_ → *c*_*x*_ in the arborescence. Once the containing relationships between groups have been established, the arborescence can be created and visualized by R igraph.

### Step 2. Dynamic creation of a co-mutation community dictionary tree

A phylogenetic tree contains smaller trees descending within its branches. A containing tree descends and branches, while within its branches a contained tree itself descends and branches. Instead of a simple pileup in a dictionary, we simulated the phylogenetic tree to leverage the hierarchical containment structure of genome groups present with the co-mutation communities to progressively build the arborescence to capture the evolution patterns of SARS-CoV-2. Specifically, we called it a dictionary tree.

#### Step 2.1. Initial dictionary tree

The initial dictionary was composed of all the co-mutation communities detected at 1^st^ week, where phylogenetic relationships were determined by their hierarchical containment in the arborescence (Supplementary Figure 5). And the arborescence structure of these communities is consistent with Qin et al. (7) using historically accumulative genomes as of 16 March 2021.

#### Step 2.2. Creation of weekly dictionary tree

Since 2^nd^ week, the dictionary trees will be built through a “union” of two trees: last week's dictionary tree and current week's co-mutation community tree (Figure 1D). Before union, similar co-mutation communities on these two trees should be first merged.

##### Step 2.2.1. Merging current week's co-mutation communities into dictionary

Co-mutation communities identified at the current week may have been included in the dictionary. While some are fresh communities composed of completely new mutations that have not been detected before, or some have common but not identical mutations in last week's dictionary. They can be a compression of, an extension of, or even partially overlap with well-established communities in the old dictionary (Supplementary Table 1). These communities were adjusted based on the principle that preserved the historical dictionary structure as much as possible where the Jaccard index was used to measure similarity of paired communities. In detail, the updating rules are: (i) a new community will be substituted by its most similar one in the dictionary if community compression occurs; (ii) a new community with an extension of at least two mutations will be progressively split into two: one corresponding to its most similar communities in the dictionary and another one for its extension; (iii) a new community will be replaced by its most similar one in the dictionary with a Jaccard similarity ≥ 0.5 (16) if partial overlap happens, or else it will be kept. All community adjustment has been listed in Supplementary Table 2.

##### Step 2.2.2. Re-creation of current week's co-mutation community tree

We re-built the co-mutation community tree at the current week using communities after adjustment according to the flowchart described in step 1.3. Before that, communities, that are identified as intermediate nodes in last week's dictionary tree and leading to those communities present at the current week, will be appended (Supplementary Figure 6).

##### Step 2.2.3. Union of last week's dictionary tree and current week's community tree

We executed the union of two trees using “union” function in R igraph. All communities (nodes) and their hierarchical relationships (edges) included in at least one tree will be preserved as part of the new dictionary tree (Figure 1D). Completely new communities which may suggest emergence of new branches are highlighted in color.

### Workflow benchmark and validation

Our dynamic surveillance framework using co-mutation network was benchmarked through monitoring major SARS-CoV-2 variants and their branches at global level. National level data from primary contributors, including South Africa, India, Brazil, Philippines, United Kingdom (UK) and United States of America (USA), were leveraged to further validate the surveillance efficiency. Considering huge fluctuation in sample size in different countries and collection weeks, distinct mutation filtration rules were utilized before genomic surveillance. Specifically, when total genomes collected across the 120 weeks were <200,000, we only kept mutations that had occurred in 10% or more of genomes with occurrences >10 in at least one sampling week. Otherwise, the same parameters were used as global surveillance. In addition, variant surveillance at national level will focus on early detection and prevalence monitoring of co-mutation communities indicating novel or rapidly circulating variants or their branches.

## Results

### Co-mutation communities capture the emergence, circulation, and extinction of SARS-CoV-2 variants

**Figure 2:**
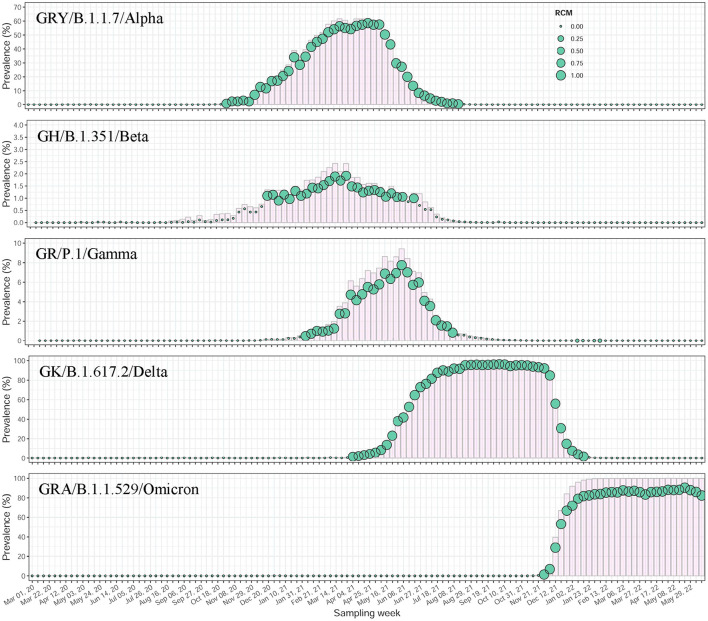
Temporal dynamics of major WHO-named SARS-CoV-2 variants identified by co-mutation communities using worldwide data. All mutations included were priorly filtered with a frequency of >1%. The scatter plots showed the prevalence changes over time of community-based variant surveillance. The circles were sized by the median of RCMs of aggregated co-mutation pairs at each sampling week. In the background, the prevalent trajectories of the variants were shown using histograms in pink.

A total of 10,246,539 SARS-CoV-2 sequences sampled between 1 March 2020 and 18 June 2022 were included in this study. These viral sequences have been distributed over 120 sampling weeks and experienced an exponential growth over time, from thousands to hundreds of thousands a week (Supplementary Figure 7). We identified 166,893 nucleotide mutations with a total of 519,230,825 mutational events from this data, but only 1,208 (0.7%) reached a frequency of >1% in at least 1 week (Supplementary Figures 8A, B), indicating a high chance of unstable mutations, or even sequencing error. The counting statistics in co-mutation discovery (see Materials and methods section) from such a giant data set showed that the co-mutation communities highly condensed viral variation information (Supplementary Table 3). These communities, illustrated by feature communities of WHO-named Alpha (B.1.1.7), Beta (B.1.351), Gamma (P.1), Delta (B.1.617.2) and Omicron (B.1.1.529) variants, demonstrated very sensitive detection in variants' emergence and concurrent growth, peaking, and decline in their epidemic, indicating strong surveillance potential (Supplementary Table 4, Supplementary Figures 9, 10). The filtration of mutations with low occurrence rate (≤1%) provided more accurate and reliable capture of viral variants' signal with a prevalence level of about 1% (Figure 2).

### Hierarchical containment between co-mutation communities reveals the phylogenetic relationships

**Figure 3:**
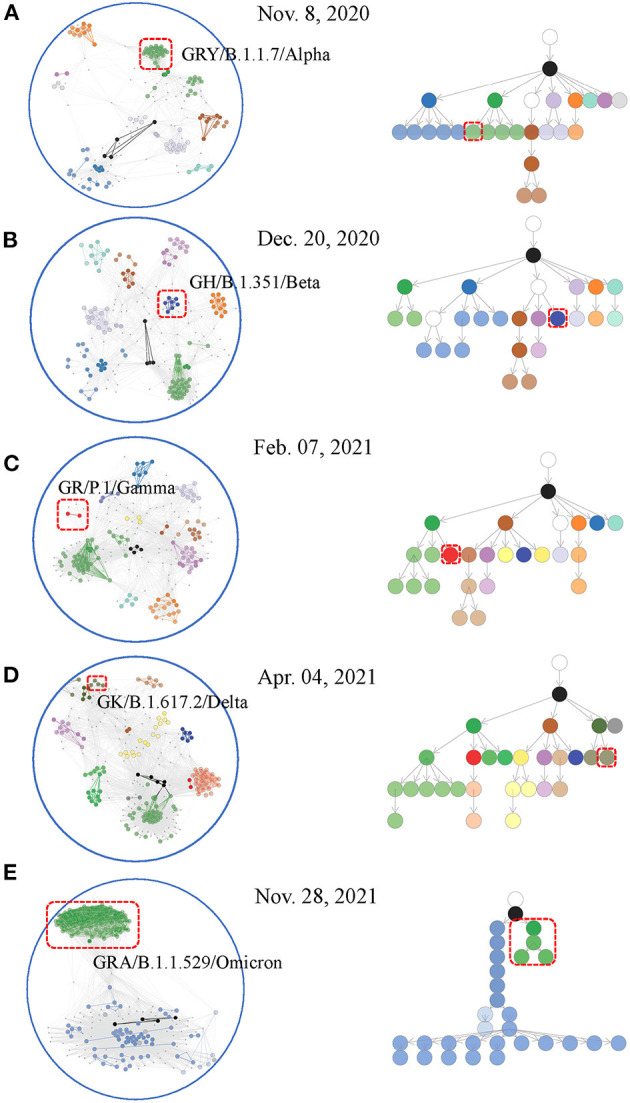
Weekly co-mutation network and co-mutation community tree for viral variant surveillance. Worldwide sequences at first detected week of major WHO-named SARS-CoV-2 variants were included for network creation [**(A–E)** left] and arborescence generation [**(A–E)** right]. Nodes (i.e., co-mutation communities) located at close branches of the arborescence were shown with the same or similar colors. Appended nodes (see **Material and methods** section) were shown in white. The same colors were designated to nodes and edges that made up the communities in the co-mutation network. Emerging communities indicating novel viral variants were highlighted with red boxes.

The affinity model was applied to each weekly data set to detect paired co-mutations. These weekly co-mutations contributed to the formulation of a co-mutation network where HoCPs gathered into groups of densely interconnected communities (see Materials and methods section). Interestingly, the co-mutation network displayed a community clustering structure (Figures 3A–E left), exemplified by the gathering of co-mutation communities into groups such that communities within groups are closer to each other. The gathered communities seemed to be connected to higher-level communities at the network center. By partitioning the viral population according to the communities' presence or not and their containing relationships, we dynamically established the hierarchical containment of the variants at different stages of the pandemic. This structure captures the hierarchical organization of these communities. These relationships were visualized using an arborescence to depict their hierarchy. This computational framework provided accurate insights on weekly epidemic communities and their branching relationships highlighting circulating SARS-CoV-2 variants (Figures 3A–E right and Supplementary Table 2). It also showed sensitive and accurate detection capability in emerging communities indicating novel evolutionary branches (Supplementary Figures 11A–E).

### Worldwide dictionary tree of co-mutation communities provides global profiles of SARS-CoV-2 variants

**Figure 4:**
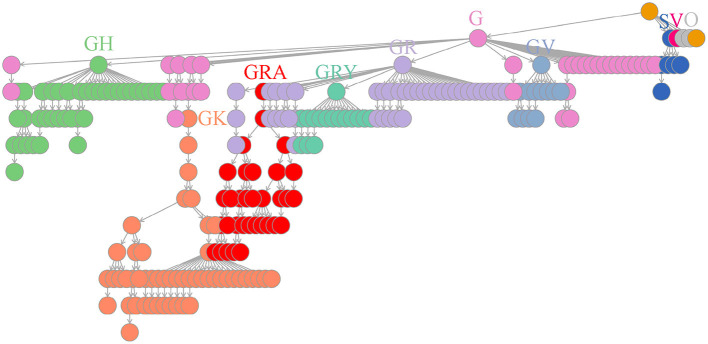
Worldwide dictionary tree comprised of co-mutation communities detected as of mid-June 2022. In total, 235 co-mutation communities were collected and piled up for arborescence creation. The branching process displayed high consistency with GISAID clades (S, V, O, G, GR, GH, GV, GK, GRY and GRA) through a comparison of weekly genome grouping using these communities or GISAID designation, which details have been listed in Supplementary Table 2.

Based on the above facts, we periodically created dictionary trees to continuously accumulate and store weekly detected co-mutation communities and their evolutionary relationships (see Materials and methods section). As of mid-June 2022, a dictionary tree comprised of 235 co-mutation communities has been built to imprint the whole evolutionary history of SARS-CoV-2 virus (Supplementary Table 5). This dictionary tree was progressively developed over 120 weeks and represented the time course of SARS-CoV-2 branching, coinciding with GISAID clades (Figure 4). Curiously, the community including the co-mutation pair of A28877T and G28878C independently appeared in different branches of Gamma (P.1) and Omicron (B.1.1.529 branches of BA.1 and BA.2), suggesting possible recombination events of these viral descendants (Supplementary Table 5).

### Dynamic co-mutation network surveillance provides early detection of SARS-CoV-2 variants

**Figure 5:**
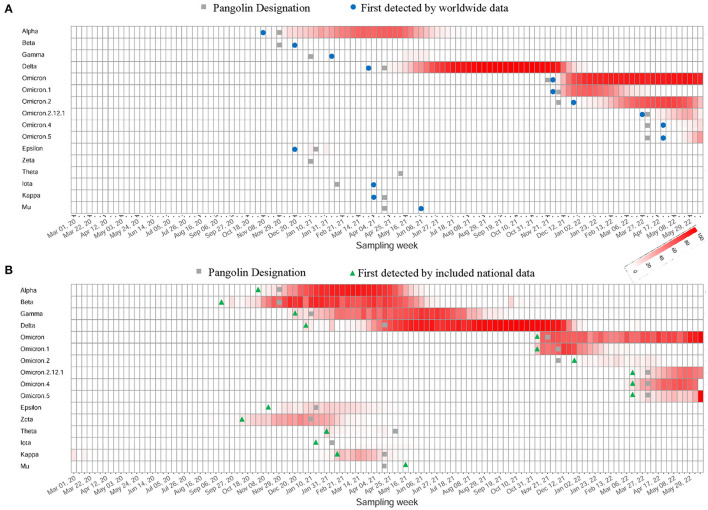
Capture of the emergence of WHO-named SARS-CoV-2 variants. **(A)** The heatmap of worldwide prevalence calculated from variants' feature co-mutation communities. Their first detected weeks were marked with different symbols depending on detection frameworks. **(B)** The heatmap of national-level prevalence of the same communities using data from country first detected. These countries include South Africa, India, Brazil, Philippines, UK or USA.

Our efficient computational framework performed SARS-CoV-2 variant surveillance through weekly tracking of the circulating co-mutation network. When novel co-mutation communities arise, our method is expected to provide timely detection at a low prevalence, identify their phylogenetic branches of emerging variants, and aid in early warning and response. We found no significant superiority for our method in detected time at global level surveillance in contrast with Pangolin's monitoring (Figure 5A and Supplementary Table 6), which may result from signal flooding due to massive data. However, it demonstrated a strong advance at national level monitoring, illustrated using data from South Africa, India, Brazil, Philippines, UK and USA (Supplementary Table 7), which exhibited significantly earlier detection of key co-mutation communities referring to major WHO-named SARS-CoV-2 variants (Figure 5B and Supplementary Table 6).

## Discussion

We developed a co-mutation network surveillance framework for dynamical nucleotide co-occurrence pattern investigation of weekly sequences and leveraged this framework to deliver an evolution and transmission monitoring of SARS-CoV-2 (Figure 1). This strategy required nothing more than weekly genomic data, enabling us to execute monitoring with only a laptop but to offer efficient surveillance of major viral variants and their branches (Figure 4). Confidence in the monitoring of spreading variants came through retrospectively evaluating multiple variants of the pandemic (Figures 2, 3) and verifying its timeliness, accuracy and reliability in detection through comparing it with Pangolin nomenclature at global and national level data sets. Our approach provided several weeks' earlier warning using national level data (Figure 5), highlighting its powerful potential in variant surveillance and public health response.

This work is a profound advancement over previous studies. First, it provides periodic co-mutation network surveillance using weekly genomic data but produces global evolutionary history through the union of weekly co-mutation communities. This method avoids the huge computational burden caused by the use of cumulative data (7). Second, the affinity model (12) was introduced for statistical discovery of weekly co-mutation pairs (either HoCPs or HeCPs), further contributing to the creation of a weekly co-mutation network. The network conglomerated HoCPs forming groups of co-mutation communities while HeCPs aided in generating community clusters that unveil novel branching patterns. This approach identifies emerging communities and their branching relationships with the latest circulating ones, indicating novel variants and their evolutionary relationships. This strategy contrasts most of previous researches that focuses on discovery of individual communities (9, 16).

Several recent efforts seek to compensate for the sensitivity and accuracy of emerging variants using phylogenetic tree to improve real-time variant surveillance. Most of these studies focus on trend survey of viral mutations (3, 17, 18) or their phenetic clustering (11, 19) but not real variant monitoring. Time-series clustering of frequency trajectories of mutations has been found to be an efficient tool in variant discovery and prediction (9, 16). Challenges arise in interpreting these results due to discrepancies in cluster features of the same variants that hinder comparisons of horizontal (between-country) or longitudinal (across-time) monitoring results. Our current work provides merging rules of co-mutation communities to overcome this problem.

The phylogenetic-tree-based methods such as Pangolin (20), Nextstrain (21), and GISAID (22) have been consistently proposed for SARS-CoV-2 variant detection and their evolution surveillance. But several challenges have been acknowledged. First, their computational complexity and statistical uncertainty in the phylogenetic construction reduce the monitoring efficiency (7). Second, their subtyping fineness either results in excess burden on variant surveillance (e.g., Pangolin with >2,000 lineages, so far) or delayed detection and communication of dangerous variants (e.g., Nextstrain with 31 clades and GISAID with 11 clades) (9). Our method gives moderate resolution of 235 variants (Supplementary Table 5) and achieves real-time variant discovery through the identification of novel co-mutation communities.

There are limits to this study. The current work provides near real-time detection of novel co-mutation communities indicating emergence of novel variants at a low prevalence but not a true appearance of previously unobserved variants. Thus, the global dictionary tree accumulated from weekly co-mutation communities recorded the major branches reaching the prevalence threshold (>1%), and could not be thought as a substitute of GISAID's global phylogeny of SARS-CoV-2. In addition, multiple consistency indexes have been introduced in our surveillance framework and their thresholds for similarity measurement are all empirical. We believe it is a trade-off between detectability and discriminability in variant monitoring. The efficacy of the empirical thresholds was verified throughout the study.

## Conclusion

In this study, a simple, explainable, and accurate approach was presented for SARS-CoV-2 variants surveillance, enabling an early detection and continuous investigation of viral variants overcoming genomic data flood and aiding in the response to the COVID-19 pandemic.

## Data availability statement

Publicly available datasets were analyzed in this study. This data can be found at: https://www.gisaid.org/.

## Author contributions

YL, YHa, and JS conceived, designed, and supervised the project. YHu and FZ collected the data. QH, HQ, and YL performed computations, analyzed the results, and drafted the manuscript. PB and JS were instrumental in reviewing and editing the manuscript. JG, JS, and YHa provided critical revision for important intellectual content. All authors contributed to the article and approved the submitted version.
