# Supplementary material for: Early detection of SARS-CoV-2 variants through dynamic co-mutation network surveillance
Source: Front Public Health. 2023 Jan 23;11:1015969. doi: 10.3389/fpubh.2023.1015969 (PMC9901361; doi:10.3389/fpubh.2023.1015969)
Supplement: Supplementary file 2 [file Data_Sheet_1.docx]

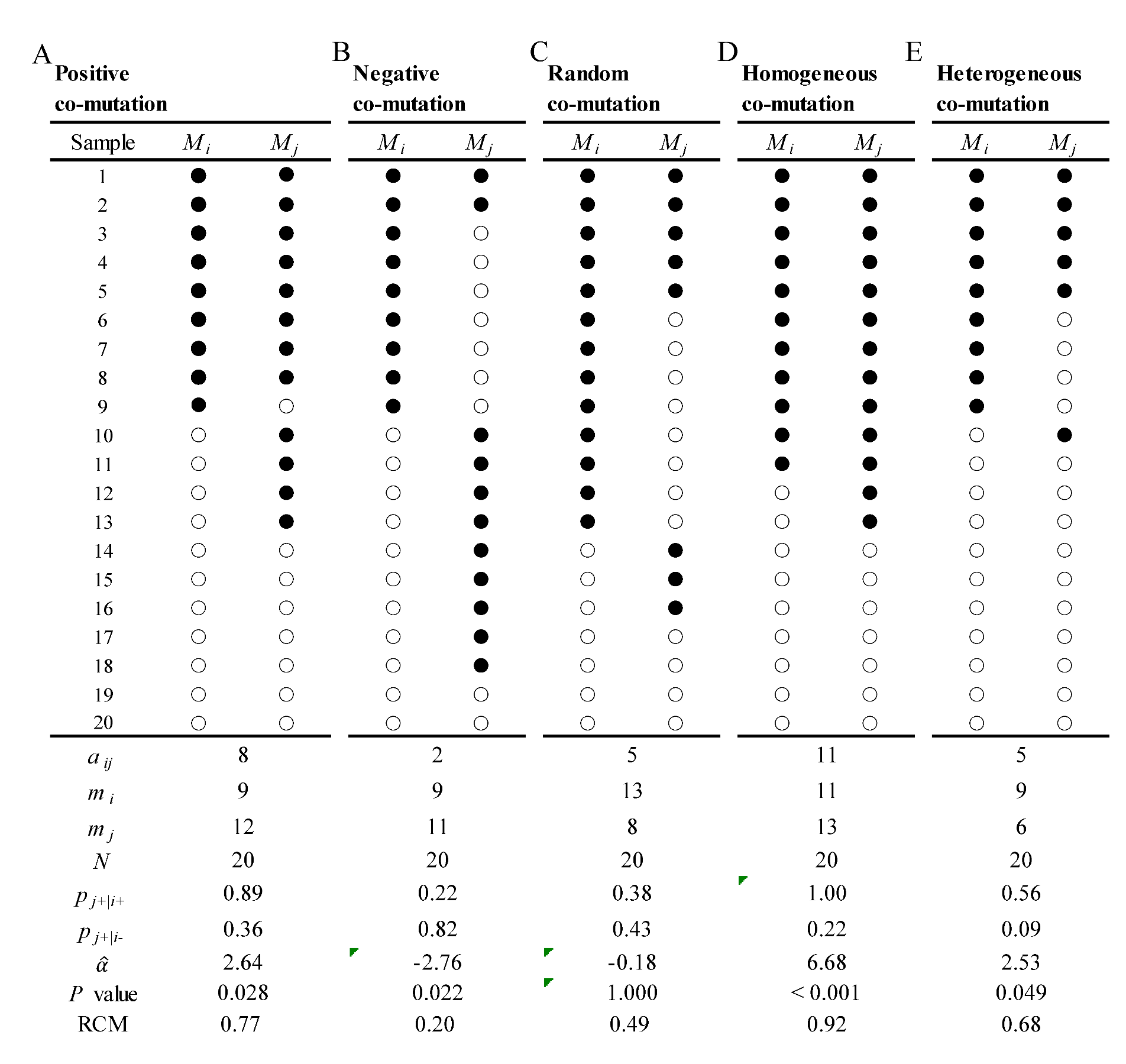


**Supplementary Figure 1.** Types of co-mutation pairs and their statistical features. (**A**) Positive co-mutation pair; (**B**) Negative co-mutation pair; (**C**) Random co-mutation; (**D**) Homogeneous co-mutation pair; (**E**) Heterogeneous co-mutation pair.


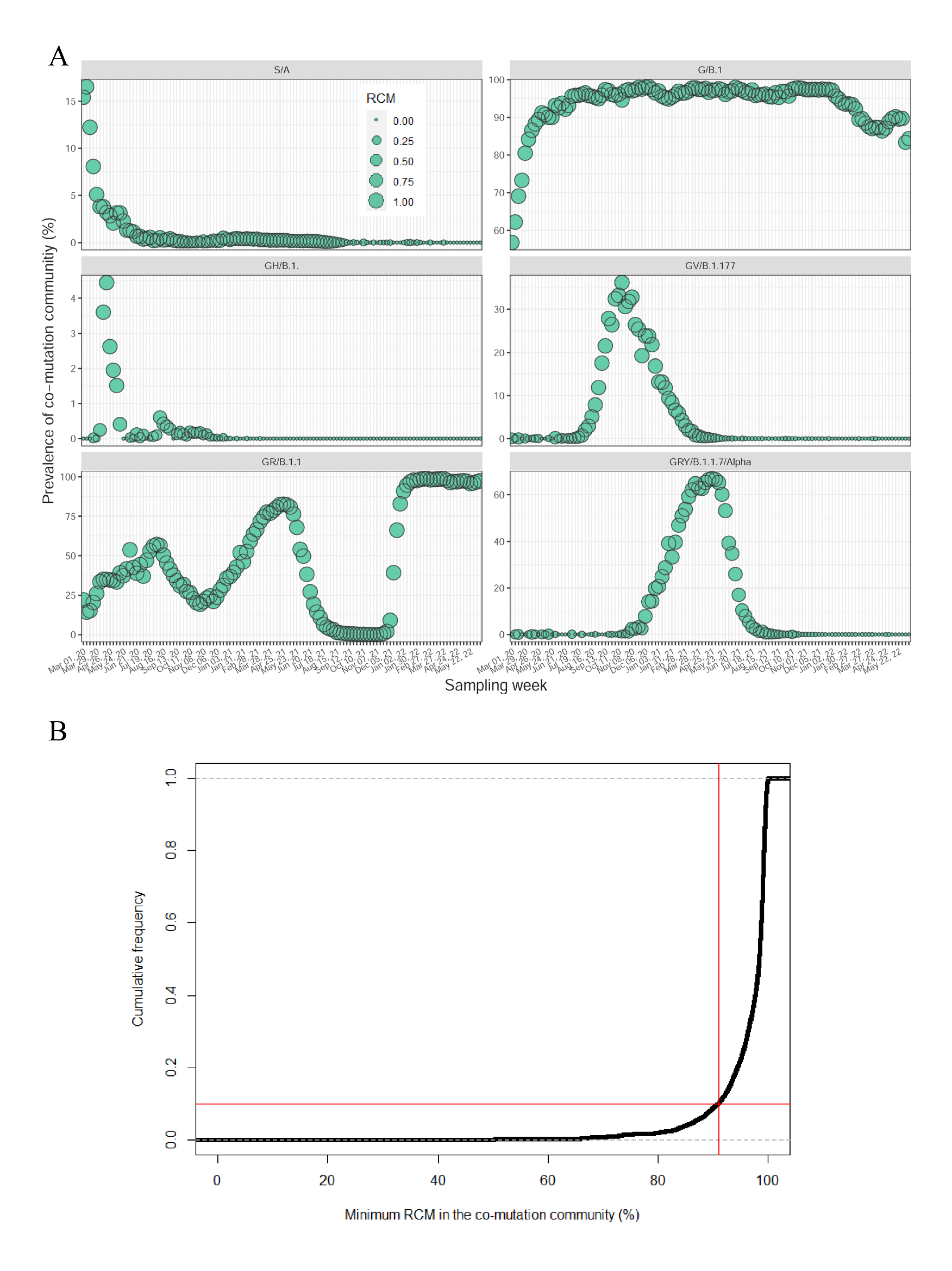


**Supplementary Figure 2.** The optimal cutoff of RCM for definition of a HoCP. Co-mutation pairs detected by Qin et al. (2021) were used as a training set. The RCM statistics for every co-mutation pair at each investigation week were calculated. Temporal dynamics of variants identified by aggregated co-mutation pairs, demonstrated by early GISAID clades of S, G, GH, GV, GR and GRY, showed strong surveillance potential when a high threshold of RCM has been reached (**A**). The cumulative distribution function of RCMs was used for threshold determination. Eventually, a 0.9 cutoff of RCM reached discovery of 90% of all co-mutation pairs (**B**). RCM - rate of the co-mutation; HoCP - a homogeneous co-mutation pair.


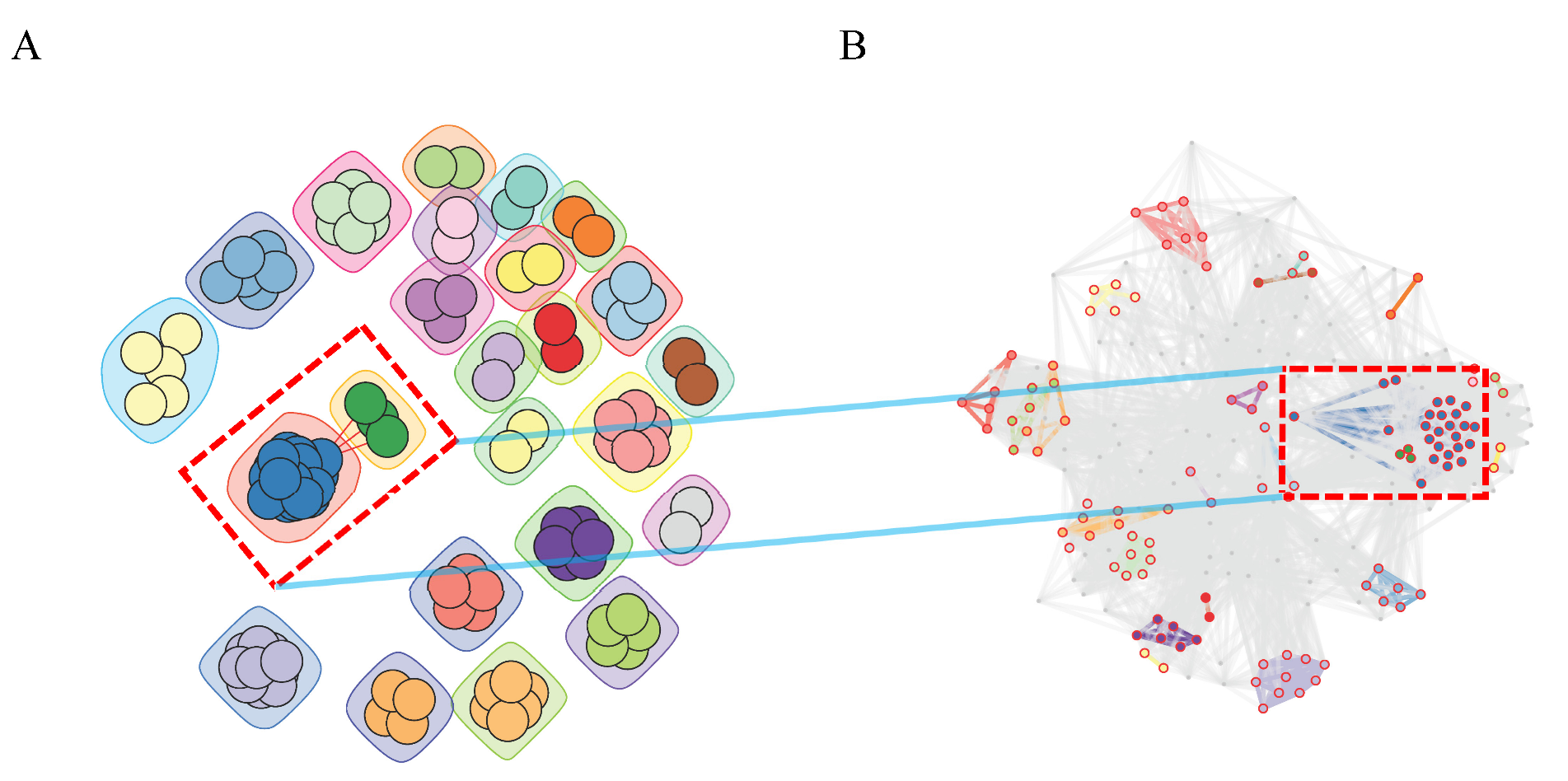


**Supplementary Figure 3.** Finer division of HoCPs using network community detection in discovery of co-mutation communities. A simple division through shared mutations, enclosed by a red box in (**A**), may miss partition details which were discovered by a community detection algorithm (**B**). HoCP - a homogeneous co-mutation pair.


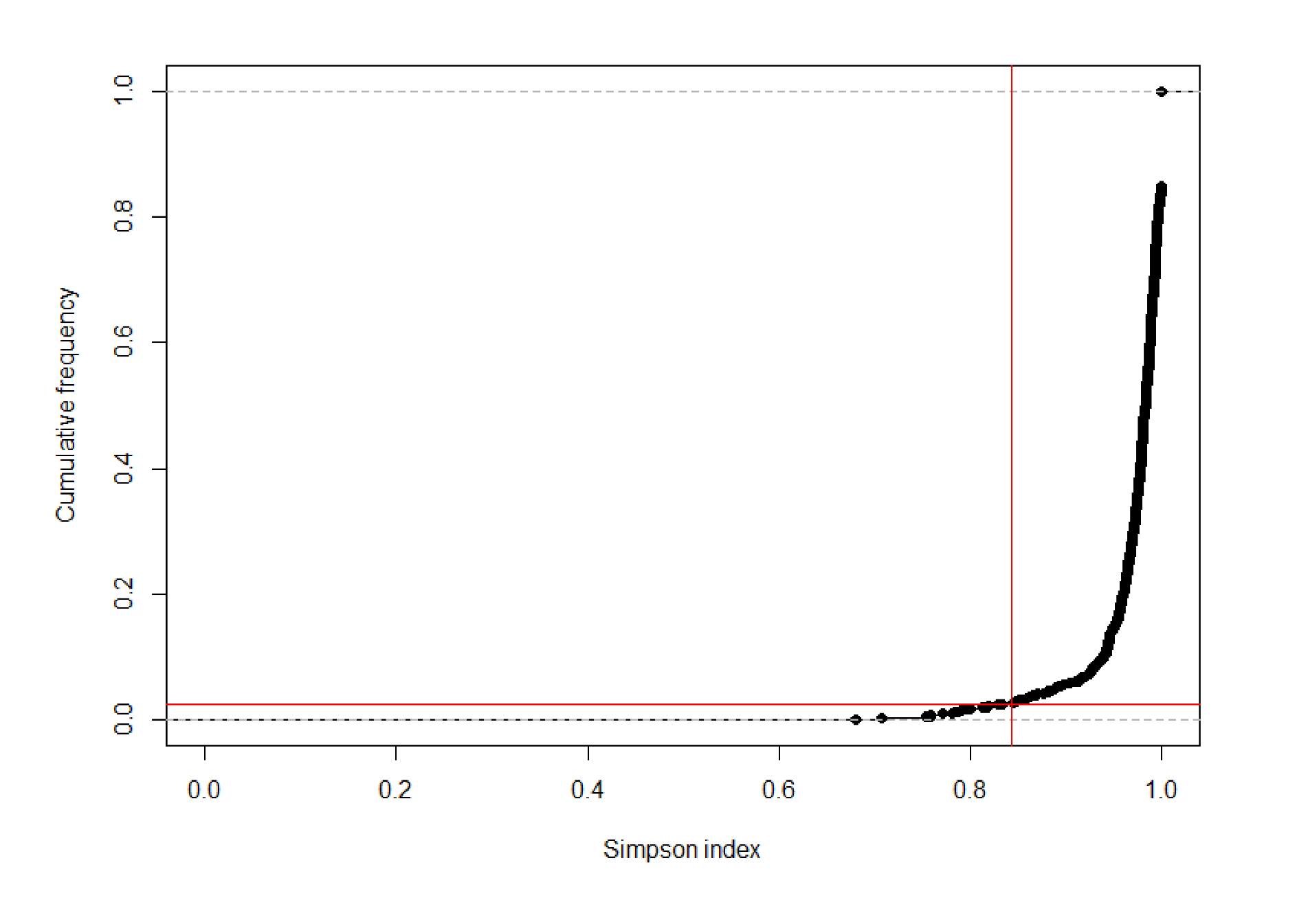


**Supplementary Figure 4.** The optimal cutoff of Simpson index for hierarchical containment establishment. Co-mutation communities detected by Qin et al. (2021) were used as a training set. The Simpson indexes for every co-mutation community at each investigation week were calculated. Their cumulative distribution function was used for threshold determination. Eventually, a 0.82 cutoff was picked up to reach a discovery of 97.5% hierarchical containment relationship.


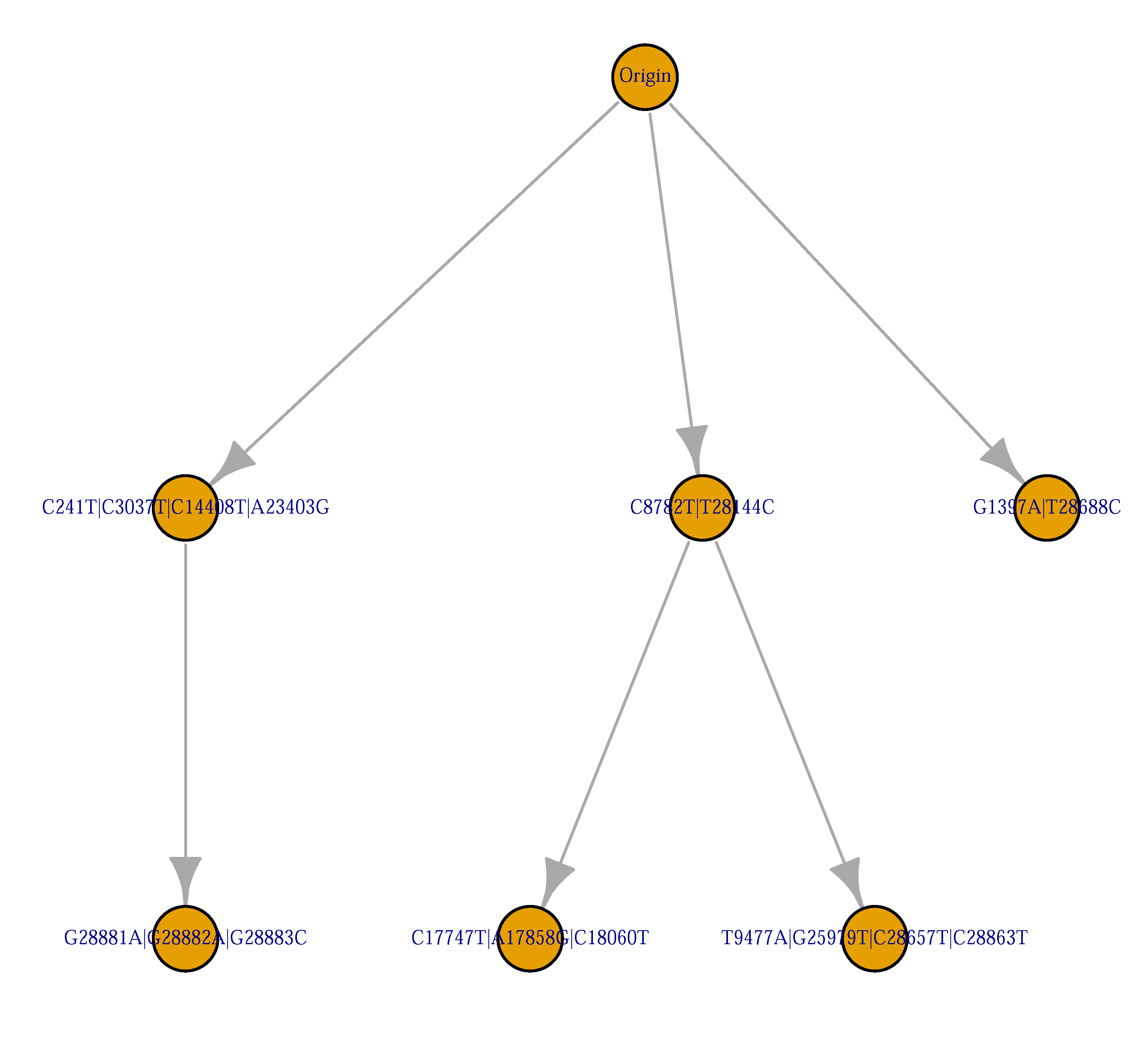


**Supplementary Figure 5.** The initial co-mutation community dictionary tree created from genomic data sampled between 1 and 7 March 2020. Six co-mutation communities were detected at that week. Their hierarchical containment were established through Simpson index using a cutoff of 0.82 and then transformed to an arborescence rooted by a complete group, which was manually assigned as ‘Origin’.


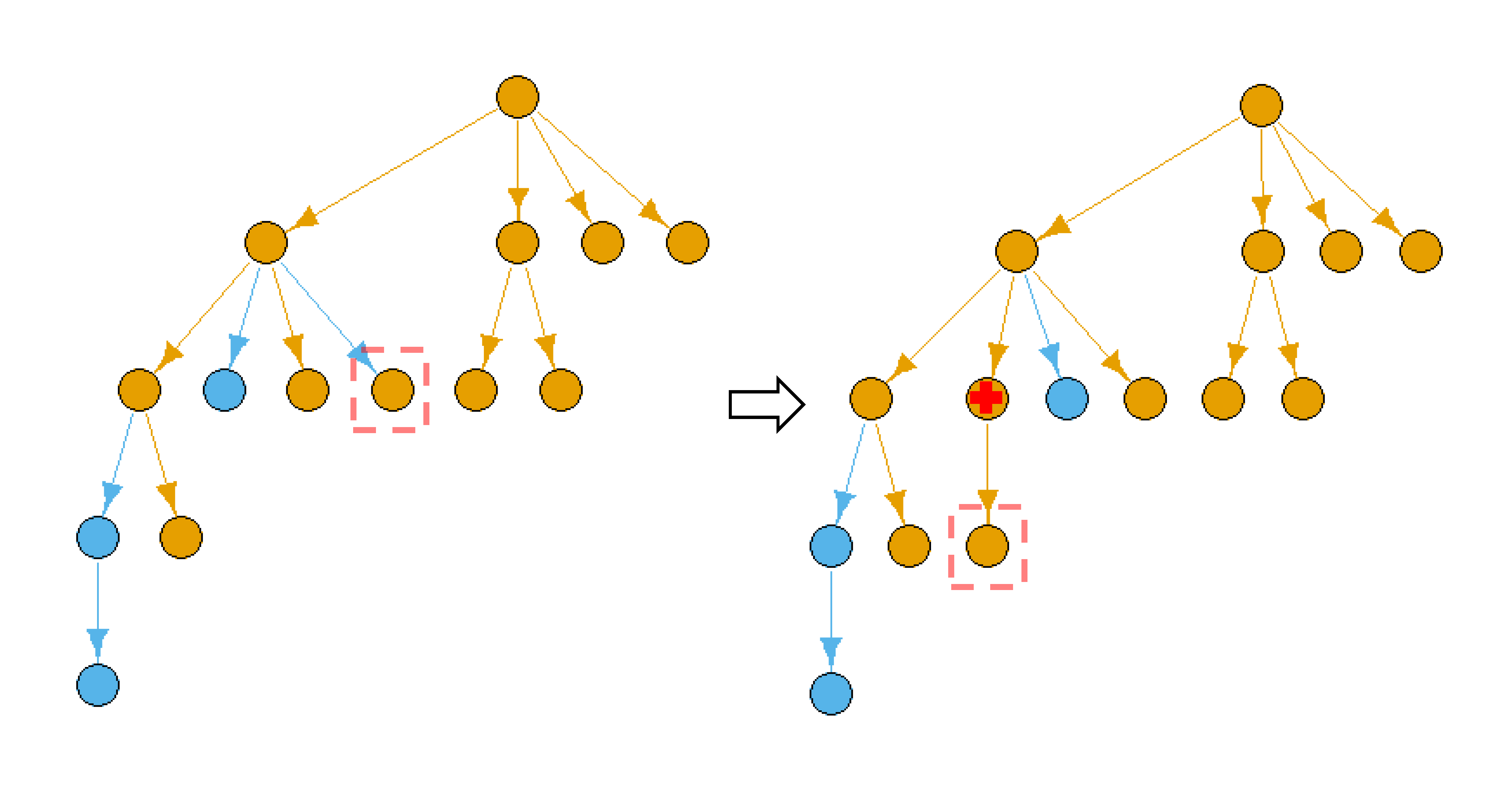


**Supplementary Figure 6.** Appendence of nodes and edges in re-creation of current week’s co-mutation community tree. There exist mistaken hierarchical relationships due to lack of detection of intermediate nodes (i.e., co-mutation communities enclosed by a red box) at the current week. The appended nodes and edges, denoted by red ‘+’, were inherited from latest dictionary tree.


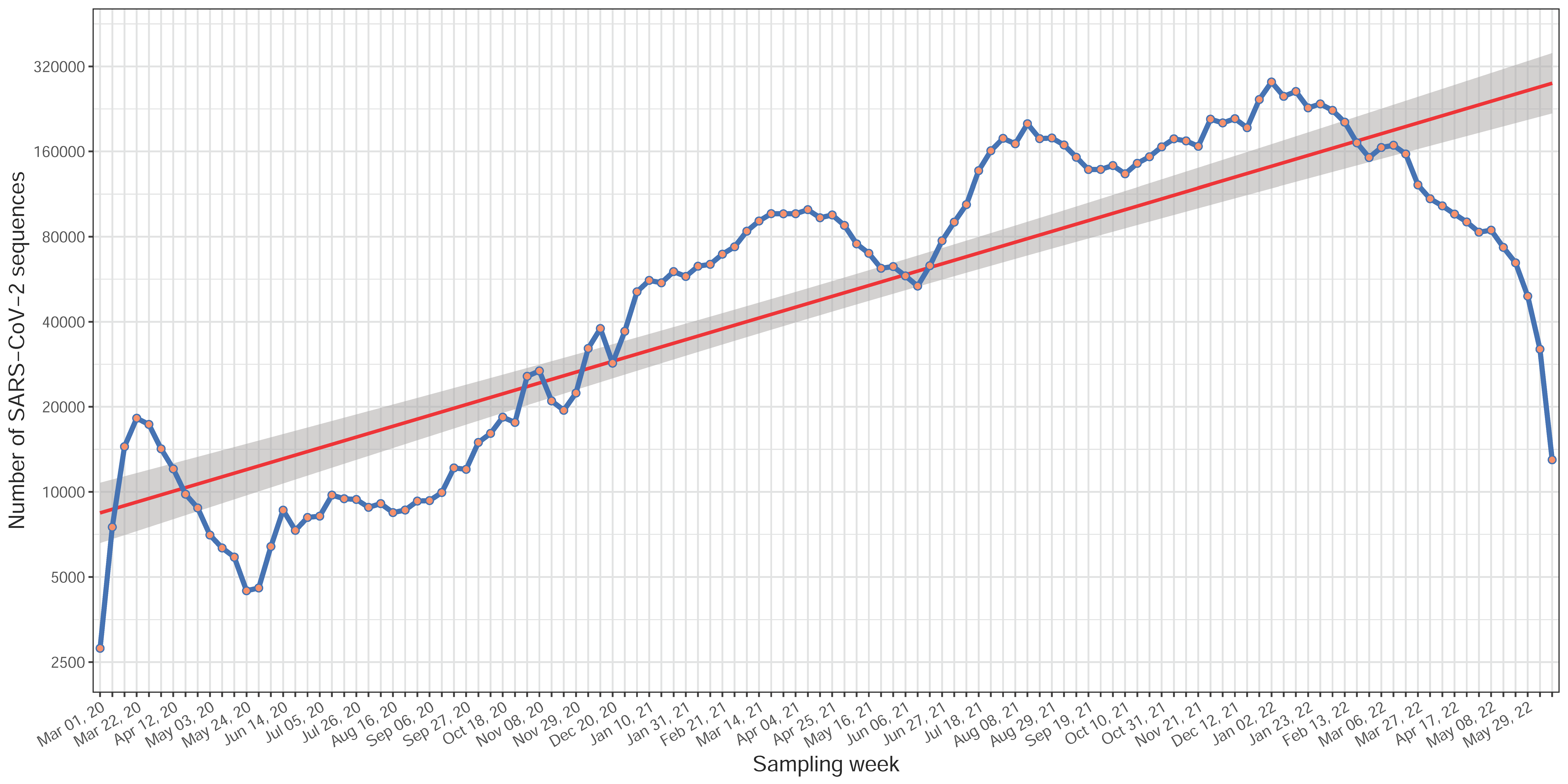


**Supplementary Figure 7.** Weekly distribution of SARS-CoV-2 genomes according to sampling time. The logarithmic linear fitting of samples by their collection week showed the genomes experienced an exponential growth over time.


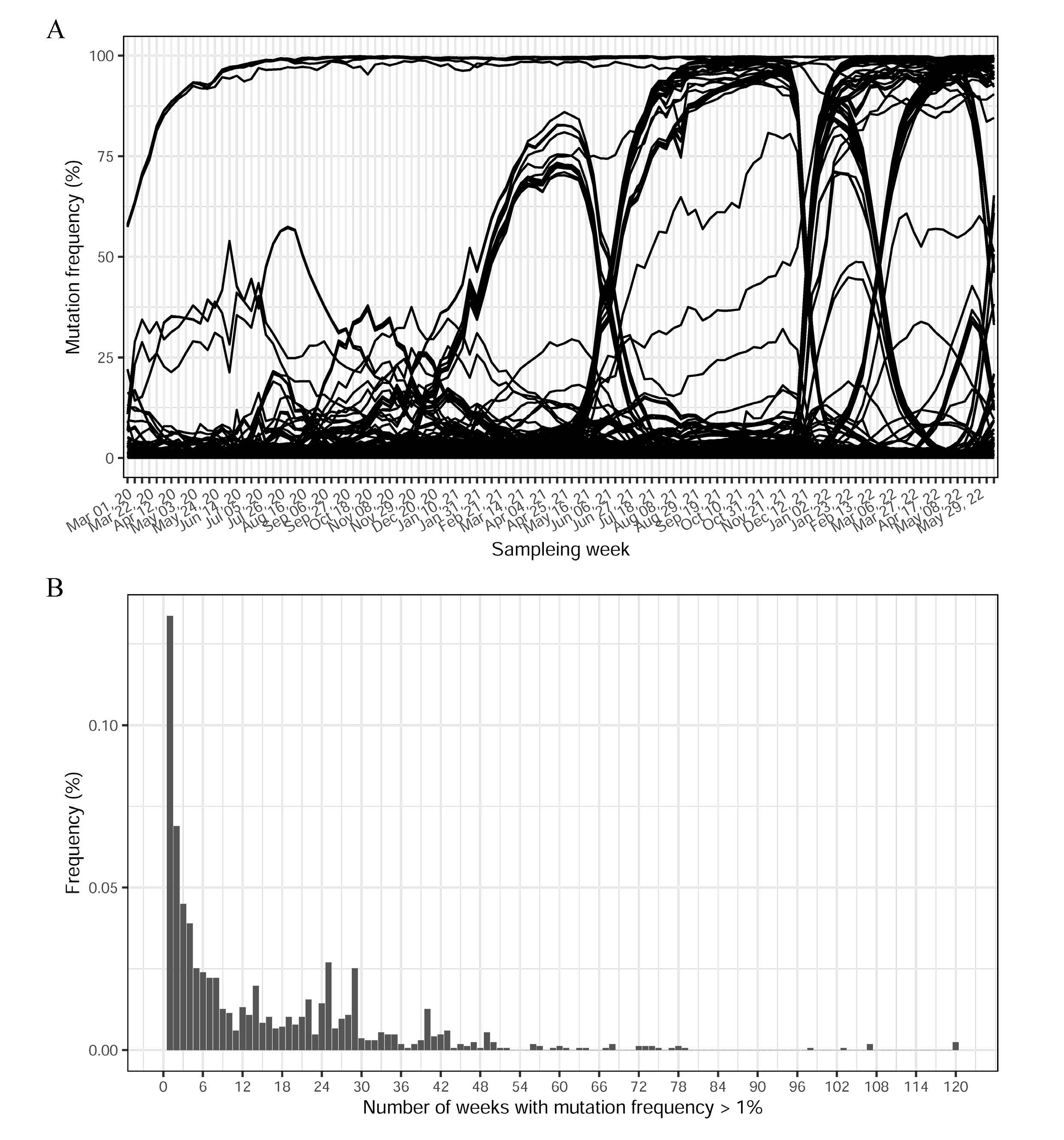


**Supplementary Figure 8.** FTM filtering rationality for worldwide data. (**A**) Frequency trajectories of mutations (FTMs). (**B**) Temporal persistence of high frequency (≥1%) FTMs. The bar-plot represents the total number of FTMs that reach a high frequency (≥1%) at different sampling weeks.


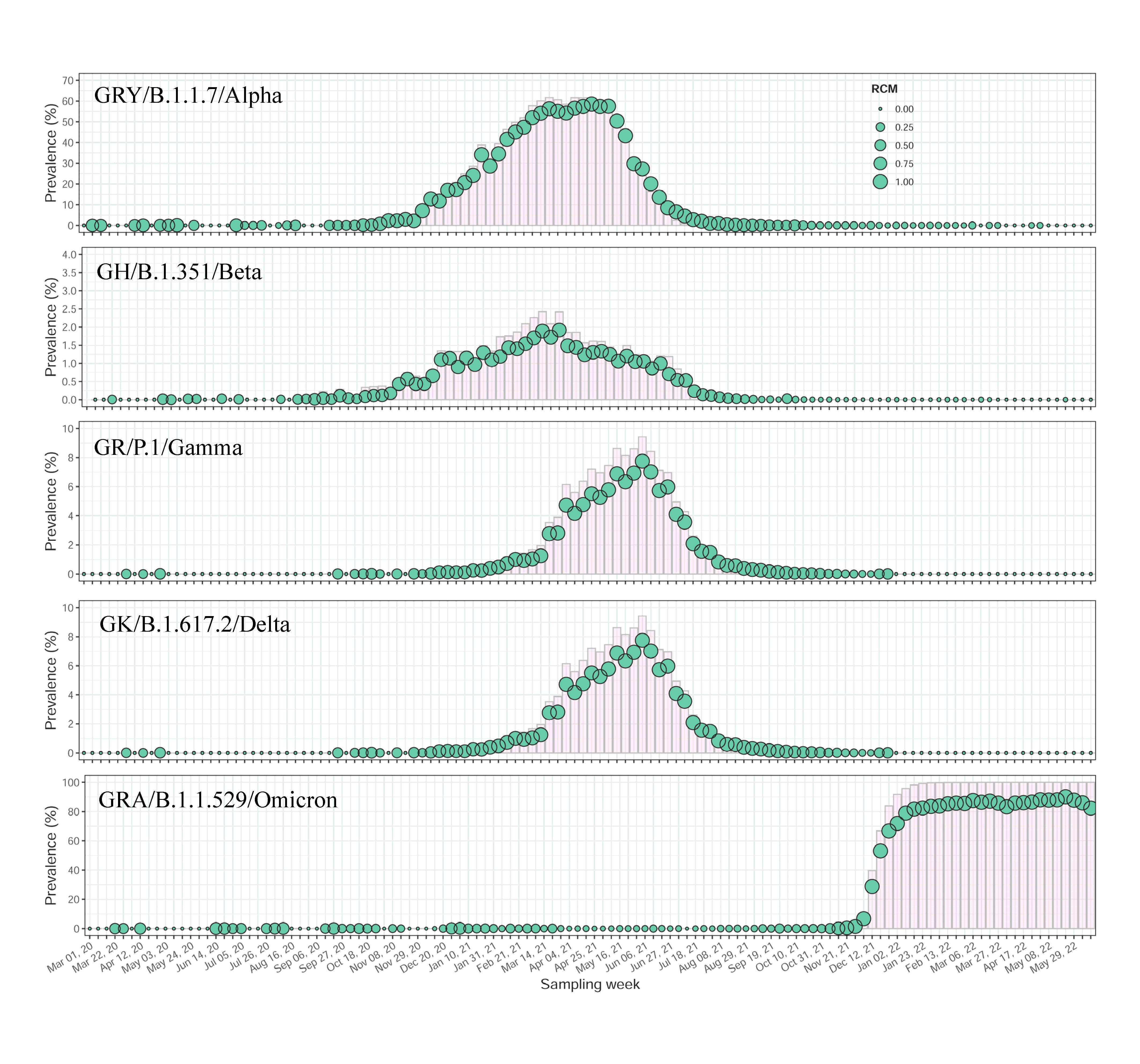


**Supplementary Figure 9.** Temporal dynamics of major WHO-named SARS-CoV-2 variants identified by aggregated co-mutation pairs (i.e., co-mutation communities). The circles were sized by the median of RCMs of aggregated co-mutation pairs at each sampling week.


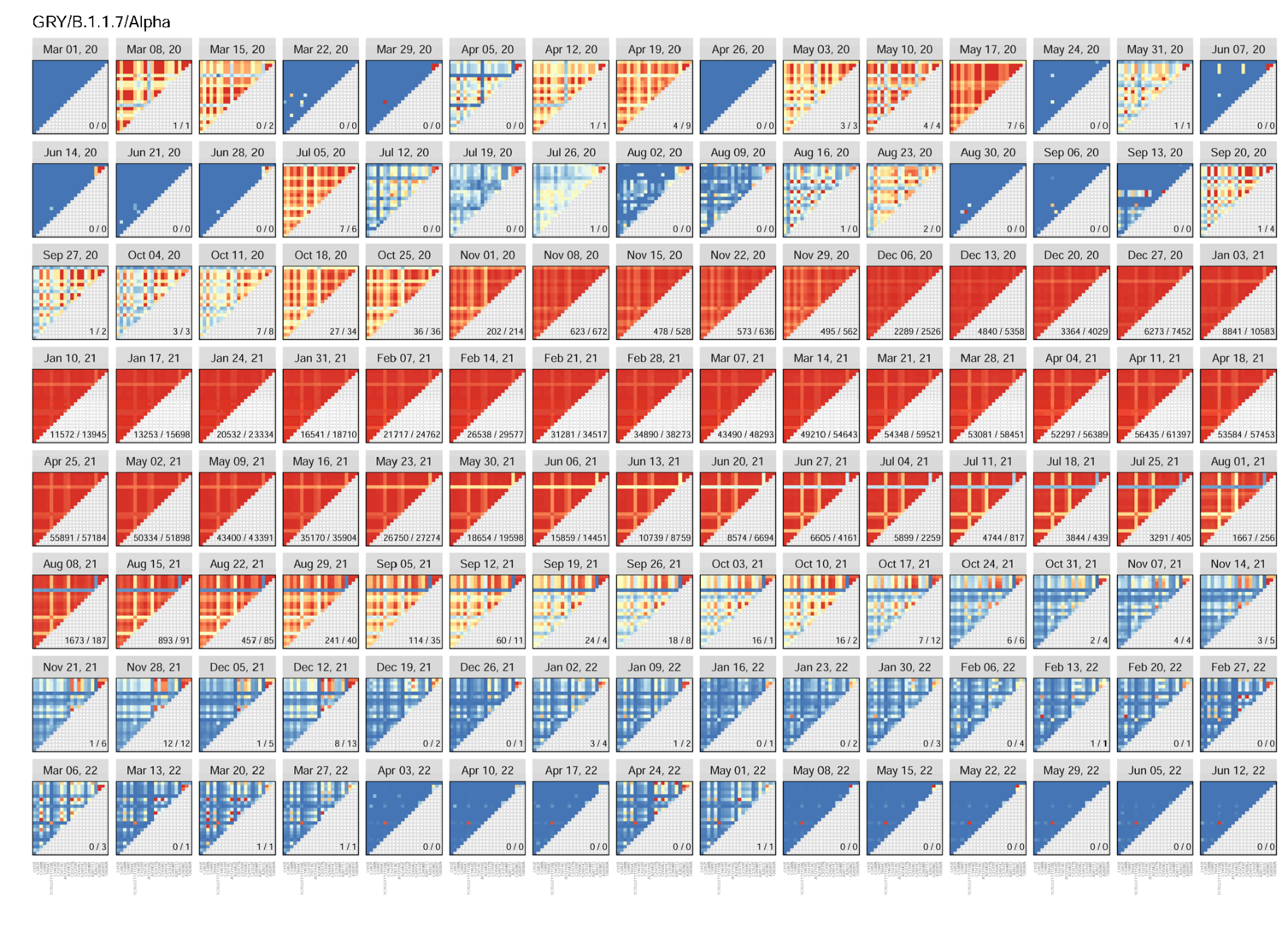


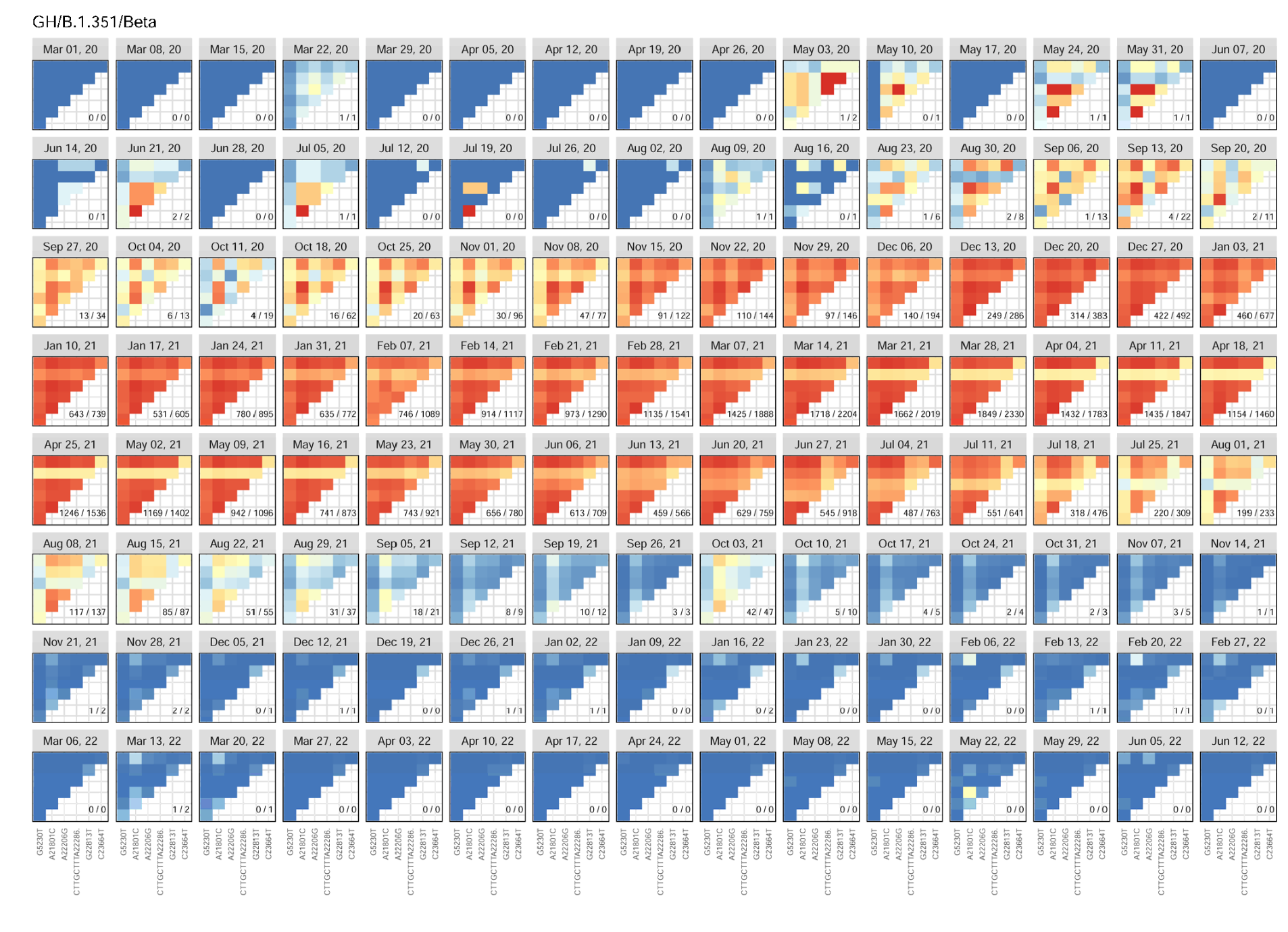


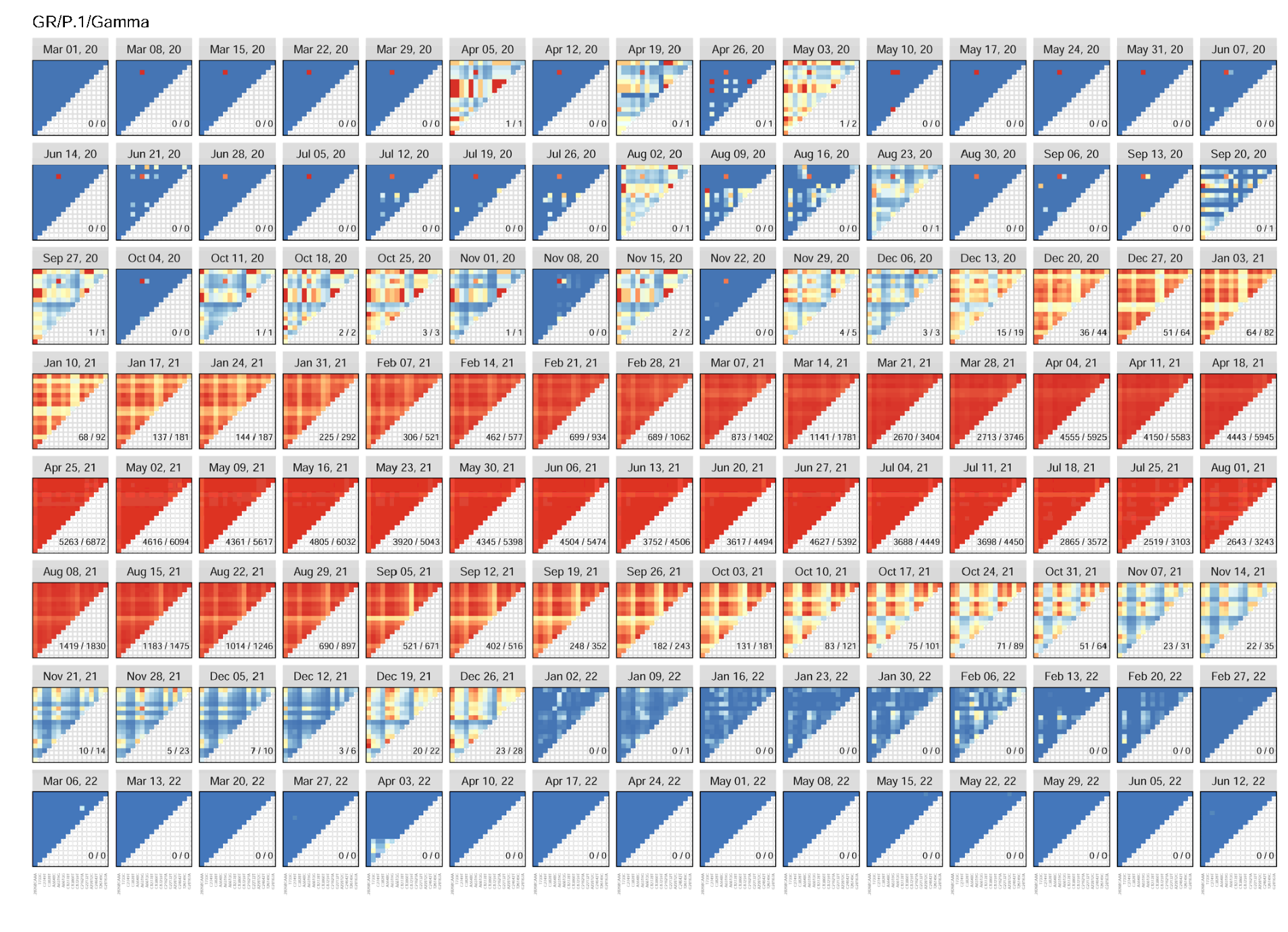


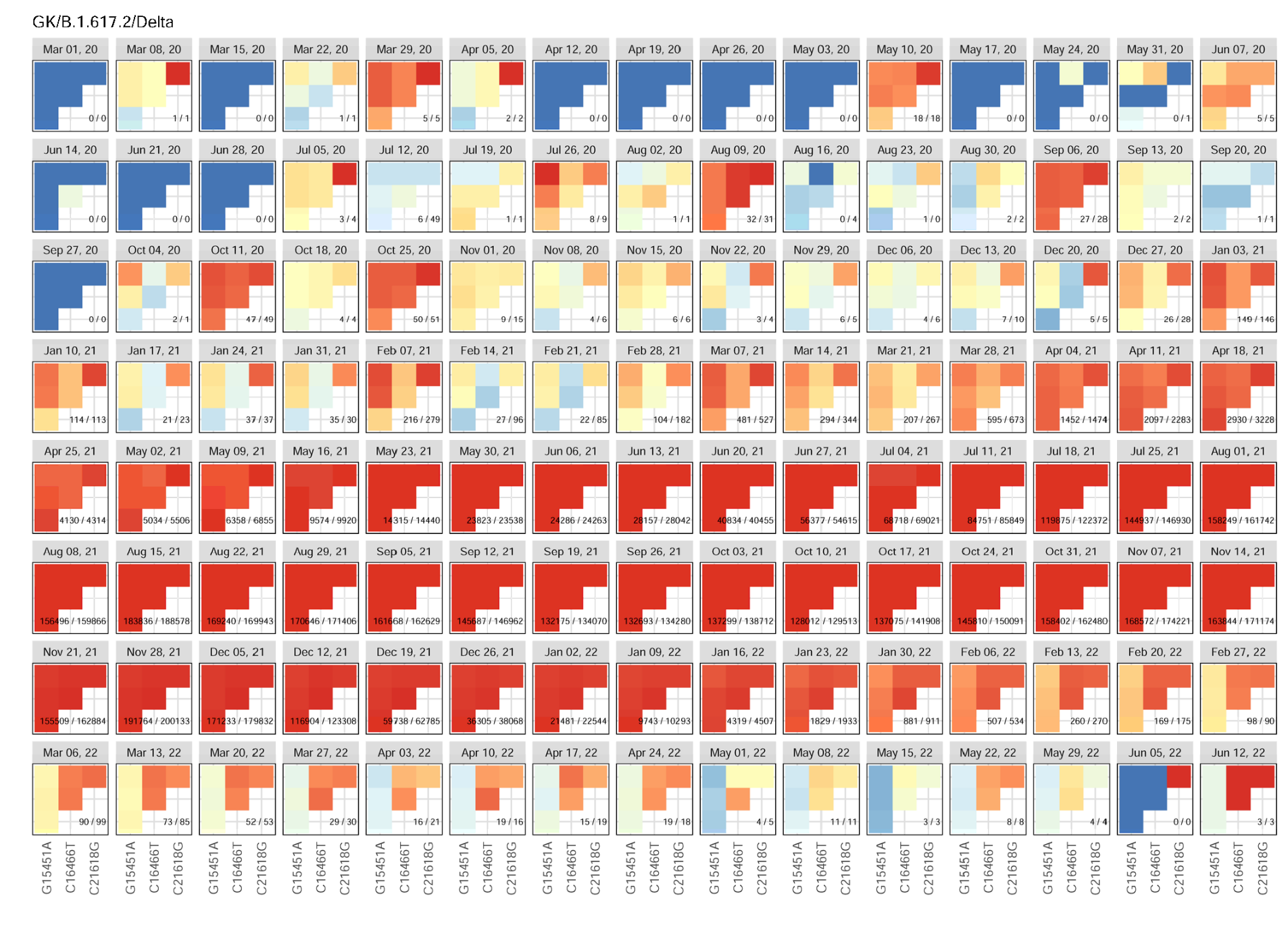


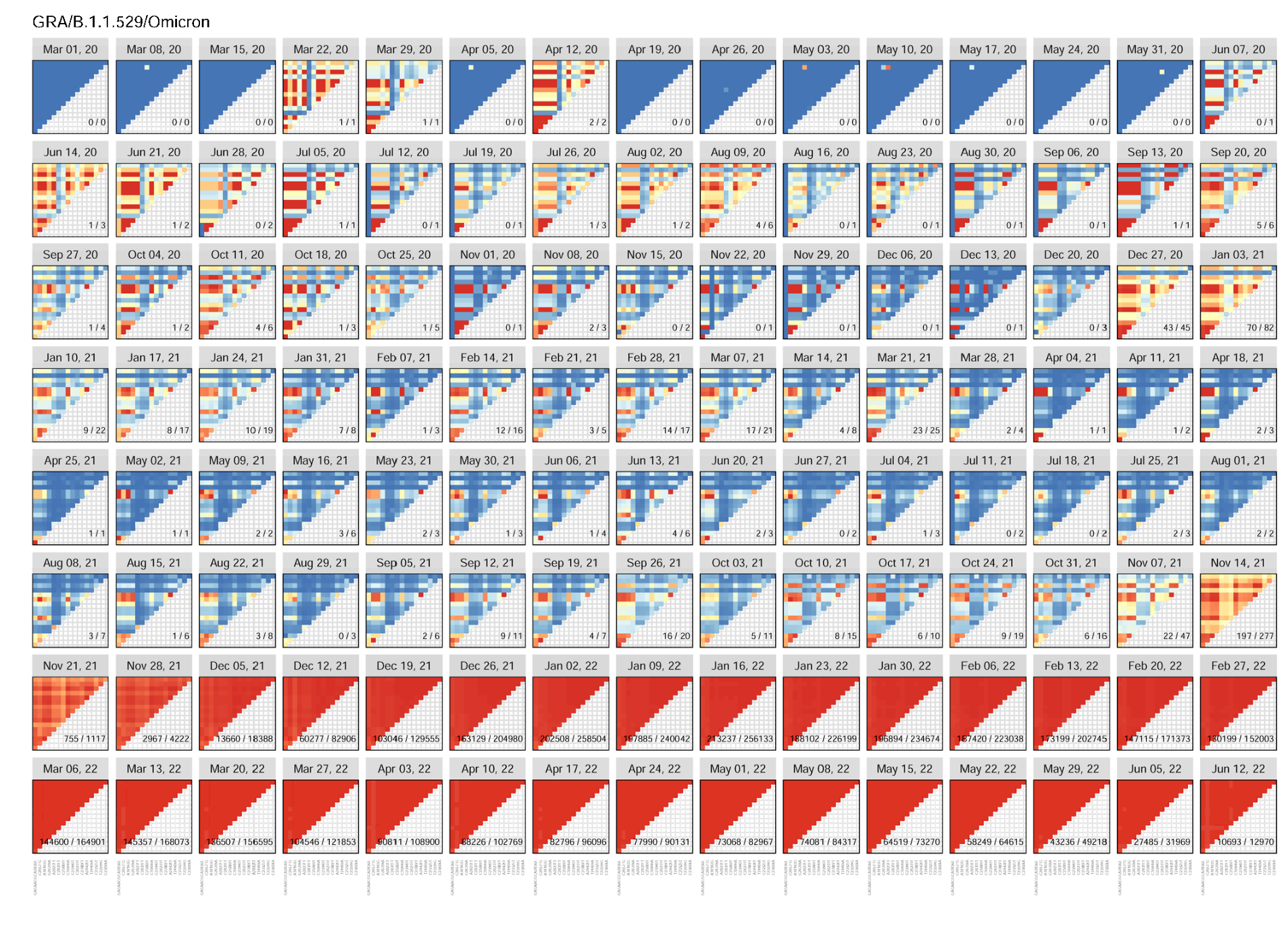


**Supplementary Figure 10.** Co-mutation signals of major WHO-named SARS-CoV-2 variants captured by co-mutation communities and shown by heatmaps changing over time. The signal intensity was measured by RCMs and weekly heatmaps were generated by these RCMs calculated from weekly sequences. RCM - rate of the co-mutation.


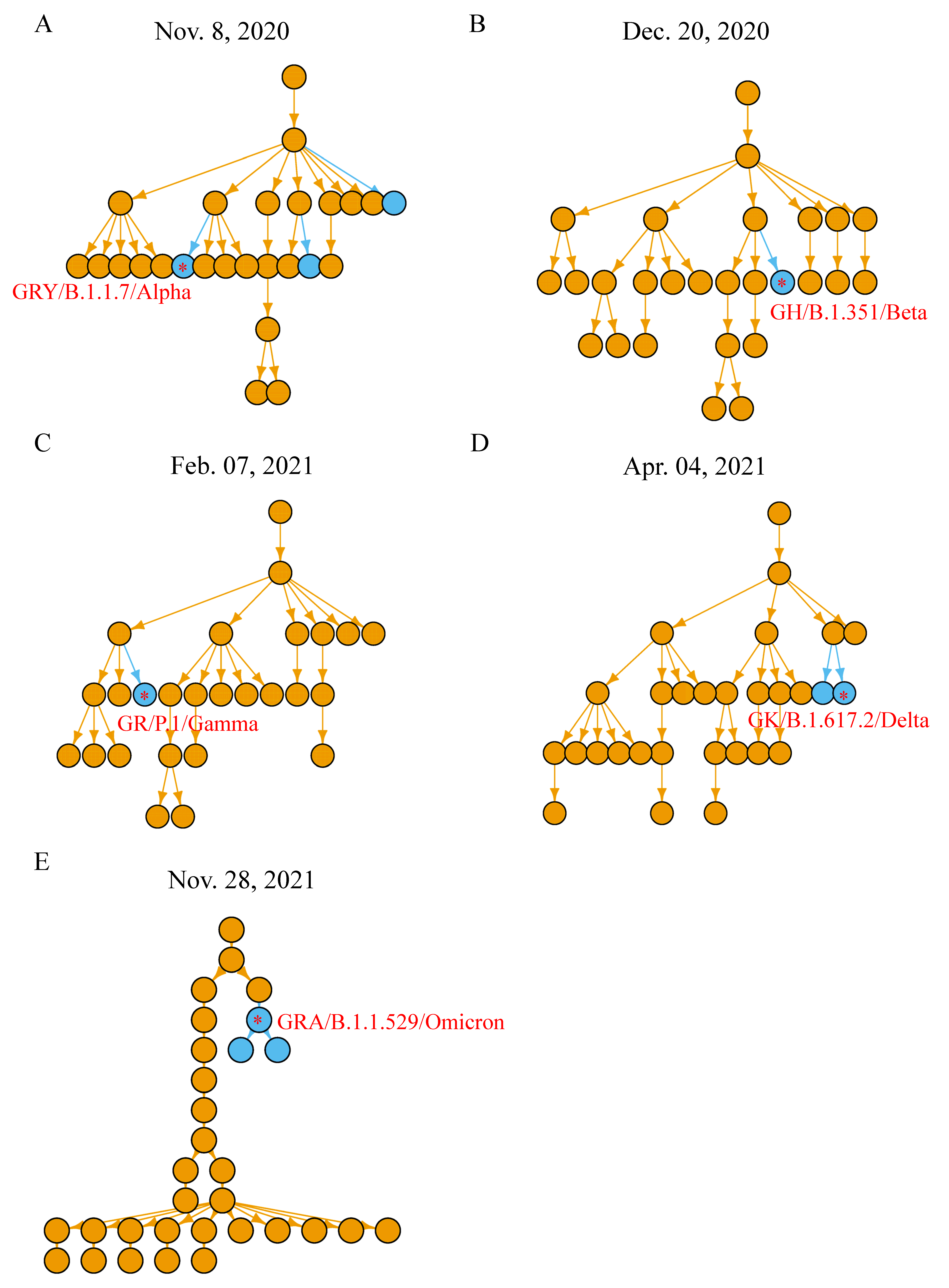


**Supplementary Figure 11.** Weekly co-mutation community tree for surveillance of emerging variants. Worldwide sequences at first detected week of major WHO-named SARS-CoV-2 variants were included for network creation and arborescence visualization. Newly detected communities were highlighted in blue.
